# Concurrent validation of the resistance intensity scale for exercise for monitoring velocity-based training with elastic bands

**DOI:** 10.1016/j.heliyon.2024.e28298

**Published:** 2024-03-21

**Authors:** Juan C. Colado, Javier Gene-Morales, Pablo Jiménez-Martínez, Angel Saez-Berlanga, Ana María Ferri-Caruana, Amador Garcia-Ramos, Jorge Flandez, Carlos Babiloni-Lopez

**Affiliations:** a Research Group in Prevention and Health in Exercise and Sport (PHES), Department of Physical Education and Sports, University of Valencia, Valencia, Spain; b ICEN Institute, Madrid, Spain; c Department of Physical Education and Sport, Faculty of Sport Sciences, University of Granada, Granada, Spain; d Department of Sports Sciences and Physical Conditioning, Faculty of Education, Universidad Católica de la Santísima Concepción, Concepción, Chile; e Institute of Education Sciences, Austral University of Chile, Ciudad de Valdivia, Chile

**Keywords:** Psychophysiological effort, Perceived effort, Rating of perceived exertion (RPE), Variable resistance training

## Abstract

The aim was to evaluate the concurrent validity and reliability of the Resistance Intensity Scale for Exercise [RISE], which uses verbal descriptors, to quantify the intensity in velocity-based training with elastic bands. Eighteen trained volunteers performed parallel squats at maximum speed at 40%, 55%, 70%, and 85%1RM in four sessions, two for familiarization and two for reliability. Each set was stopped at a 10% intra-set velocity loss. Participants reported the perceived effort (easy-low-moderate-hard-maximal) at the first and last repetition. The concurrent validation was conducted with external load (i.e., mean propulsive velocity, weight, repetitions, and maximum power) and internal load parameters (i.e., heart rate). Participants' relative strength was calculated to assess its influence on the dependent variable. Acceptable concurrent validity and reliability (ICC>0.77, CV<21%) were observed, with the perceived effort being appropriate to differentiate between intensities and not being influenced by the participants’ relative strength (*p* = 0.88). A categorical linear regression showed significant (*p* < 0.001) associations between the RISE scores and the weight, repetitions, and mean propulsive velocity (*r* = 0.43–0.63). The findings certify the usefulness of the perceived exertion for quantifying the intensity during velocity-based training with elastic bands. The perceived exertion of the first and last repetition favors a proper dosage of the training load.

## 1 Introduction

Lack of time and difficulty in accessing adequate traditional resistance training facilities (e.g., commercial gyms with free weights and machines) are the main barriers for people to engage in resistance training programs [1]. To overcome these barriers, there are time-efficient, accessible strategies (e.g., elastic bands). Resistance training with elastic bands provides comparable strength and health-related gains compared to traditional methods (e.g., free weights, machines) in populations with different training levels [2]. Additionally, elastic bands are particularly well suited for home-based resistance training interventions [3,4]. However, to ensure the effectiveness and security of resistance training with elastic bands, the intensity must be monitored [5]. For such purposes, rating of perceived exertion (RPE), performance-related (e.g., kilograms, velocity), or physiological (e.g., heart rate) parameters are among the most useful [6].

Different RPE scales (i.e., OMNI-RES, repetitions in reserve [RIR], or Borg scale) have been employed as tools to quantify training intensities, dosage, and progress [[7], [8], [9]]. RPE scales are based on the relationship between the increase in intensity during physical exercise and the perceptual, physiological, and performance responses [6]. RPE scales designed with numerical descriptors are commonly seen. However, some people (e.g., with cognitive impairment) struggle to associate numbers with words when trying to describe feelings related to exercise [10]. Therefore, RPE scales with verbal descriptors (e.g., Resistance Intensity Scale for Exercise [RISE]) might be more suitable for some populations. Although further studies are needed. The RISE also serves to characterize upper- or lower-body exercises and can represent the overall body or regional fatigue [11]. The RISE has been one of the first RPE scales validated to quantify the intensity during exercises for upper- and lower-extremities with elastic bands [12].

Regarding the resistance training methodology, velocity-based training (VBT) offers several advantages compared to traditional approaches to quantify the training intensity (e.g., one repetition maximum [1RM]). For instance, measuring the concentric velocity of the barbell is time-efficient [13], reliable (i.e., stable values between sessions) [14], and valid (R^2^ = 0.91–0.95) to estimate the 1RM [15]. Thus, VBT allows an easy assessment of the optimal (i.e., programmed) load or resistance to use during training sessions [16]. Therefore, the identification of the RPE during the first repetition of a set may help to quickly adapt the weight/resistance according to the training session aim. Additionally, VBT at ≤ 10% of intra-set velocity loss favors the optimization of the neuromuscular training stimulus [17], increasing muscle strength and reducing neuromuscular fatigue and chronic muscular damage [18]. In this sense, the question arises as to whether the identification of a specific RPE associated with an intra-set velocity loss of 10% may help to avoid undue fatigue. Another key parameter to consider during VBT is the relative strength ratio of the exerciser, which is calculated as 1RM load/body mass. This parameter shows significant effects on VBT outcomes, such as MPV loss [19,20]. Nevertheless, nothing is known about whether relative strength can influence the perceived effort (i.e., RISE) during VBT.

Previous studies have used the RPE to estimate the correct dosage of the intensity in VBT with elastic bands [21,22]. Conversely, the RPE for VBT with elastic bands did not undergo acute validation and, from a global point of view, this could lead to conceptual errors [12]. Specifically, previous validations of the RPE for tracking the intensity while training with elastic resistance have used a traditional approach (i.e., moderate-controlled velocity of execution and RPE reported right at the end of each set). At this point, it can be highlighted that the RPE obtained through numerical descriptors (0–10, OMNI-Resistance Exercise Scale for elastic bands) in the first and last repetition has recently been presented as useful for monitoring the training load (i.e., movement velocity, power, weight used, number of repetitions) during VBT with elastic bands [23]. However, as far as we are aware, no previous studies have applied the RPE of the first and last repetition through word descriptors (easy-maximal, Resistance Intensity Scale for Exercise) to quantify the training load during VBT with elastic bands.

The aims were (i) to assess the concurrent validity and (ii) between-session reliability of the Resistance Intensity Scale for Exercise for monitoring the intensity in squats at maximum velocity with elastic bands. We also evaluated the influence of the relative strength of participants on the dependent variables. Additionally, it was aimed (iii) to study the potential of the Resistance Intensity Scale for Exercise scores to predict external load parameters at different loads (i.e., 40–85%1RM). It was hypothesized that (i) the Resistance Intensity Scale for Exercise is valid to quantify the intensity during VBT with elastic bands, with the relative strength of the participants not influencing the chosen score on the Resistance Intensity Scale for Exercise scores. Additionally, it was also hypothesized that (ii) the Resistance Intensity Scale for Exercise would present good reliability scores and (iii) significantly predict the external load variables analyzed.

## 2 Materials and methods

### 2.1 Design

This research is part of a larger project (approved by the Ethics Committee of the University of Valencia; code: H20190325095509; previously published studies [23,24]) that aims to analyze the outcomes of using elastic bands, instead of weight plates, to load the bar in the parallel squat at maximum velocity of execution and validate the RISE of the first and last repetition.

A quasi-experimental design was employed to validate the RISE for monitoring the intensity in squats performed at maximum velocity with elastic bands. External (i.e., mean propulsive velocity [MPV], number of repetitions, weight used, maximum power [P_MAX_]) and internal (i.e., heart rate) load variables for the concurrent validation were used. All procedures were applied according to the Declaration of Helsinki. The participants gave informed consent and were free to leave the study.

### 2.2 Participants

We determined the sample size using G* Power 3.1 software [25], to reduce the probability of type II error [26]. The analysis configuration was: F-tests/ANOVA: repeated measures/mixed design. A sample size of 18 volunteers was required to meet a statistical power of 0.80, α = 0.05, a correlation coefficient of 0.50, nonsphericity correction of 1, and an effect size of 0.60, based on previous studies from our research group [23,27].

Therefore, twenty-one subjects volunteered to participate. However, only 18 physically active participants completed all the required sessions (twelve men and six women; age: 23.7 ± 3.4 years; body mass index: 23.3 ± 2.6 kg/m2; body fat percentage: 12.4 ± 3.8%; 1RM: 104.8 ± 27.7 kg; ratio 1RM-bodyweight [relative strength]: 1.5 ± 0.2). The participants answered the Global Physical Activity Questionnaire (GPAQ). All volunteers reported moderate (≥600 to <3000 METs, n = 8) and high (≥3000 METs, n = 10) weekly physical activity levels [28]. With the purpose of not altering their perceived exertion during the whole training [29], only participants with previous experience in resistance training (minimum of six months) and including the squat exercise in their training were eligible. Participants were excluded if any type of musculoskeletal injury was suffered one year before the participation [30]. The consumption of ergogenic or nutritional aids was not allowed throughout the study or during the period of participation. Participants were asked not to exercise the lower limbs at high intensities ≥24 h before each session and to sleep ≥8 h.

### 2.3 Procedures

The anthropometric measurements, evaluation of the 1RM load, and familiarization of the participants with the RISE and testing procedures were conducted in two previous sessions. Participants were advised to wear light clothes and remove their shoes for the anthropometric measurements. A stadiometer (IP0955, Invicta Plastics Limited, Leicester, UK; precision 0.01 cm) was used to measure the height. The participants, with the back close to the stadiometer, took a deep inhalation at the time of the measurement, keeping the Frankfort plane. A bioelectrical impedance scale (Tanita model BF-350, Tokyo, Japan; precision 0.01 kg) was used to measure weight and body fat %. The participants stood barefoot on the bio-impedance scale, and each foot was placed on the corresponding pairs of electrodes with arms extended. Additionally, the squat range of movement for each participant was standardized using a goniometer [31]. The general warm-up consisted of 10 repetitions of shoulder, hip, knee, and ankle dynamic mobility in each plane of movement possible for each joint. Followed by two sets of another 10 repetitions of skipping, jumping jacks, and lateral jumps with both feet. In the specific warm-up, a set of 15 squats and 10 lunges with each leg with the own body weight were performed. Finally, there was an approximation set before the experimental protocol, performing 20 repetitions with the bar (20 kg). After the 10-min warm-up, the 1RM was indirectly calculated (see Section 1RM *Calculation*). Thereafter, participants randomly (i.e., sealed envelopes) executed four sets at 40%, 55%, 70%, and 85% 1RM. These sets were designed for participants to gain more experience with the RISE and squat protocol. This was performed in both familiarization sessions.

Seventy-two hours later, we conducted the two experimental sessions. In both experimental sessions, four sets at 40%, 55%, 70%, and 85% 1RM using elastic bands to load the bar were performed in random order, as previously suggested [23,27]. Moderate (40–55% 1RM) and heavy (70–85% 1RM) loads were used to perform the parallel squat since the higher mean power is expected to be in this range of the 1RM continuum [32]. Moreover, a difference as small as 15% 1RM was employed to know if RISE is more sensitive than what had been previously studied [12]. Five minutes of rest were standardized between each set. Each set was finished when a minimum of 10% intra-set velocity loss was achieved. Meanwhile, MPV and P_MAX_ of the first and last repetition were recorded. Each participant verbally mentioned the RISE word descriptor after the first and last repetition. The scales were placed in front of the participants and were visible at all times. Finally, we measured heart rate, kilograms at the standing position, and number of repetitions performed when the set was finished.

### 2.4 Variables and instruments

#### 2.4.1 Exercise protocol

**Fig. 1:**
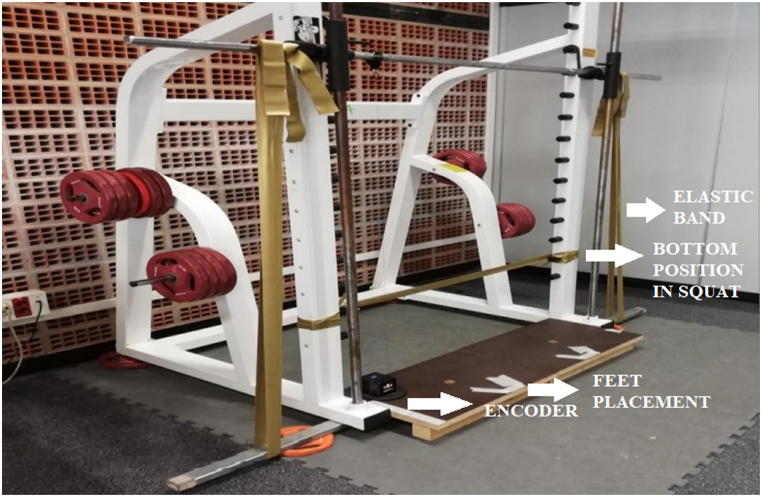
Setting to perform the squats with elastic bands.

A guided Smith machine (Powerline, PSM14X, Body-Solid, Chicago, IL, USA) was chosen for the parallel squat because the linear position transducer used in our study (see Section *Movement Velocity and Maximum Power*) shows more reliable results with guided movements [14,33]. Elastic bands (CLX bands, TheraBand® Hygenic Corporation, Akron, OH, USA) to load the bar were used. We used a digital scale (Salter, Model 9179 SV3R, Barcelona, Spain; precision of 50 g) and a strut with a range of 95–170 cm (Piher, Model 30011, Logroño, Spain) to measure the weight generated by the bands. The tension of the elastic bands was adjusted at each participant's upright position based on the kilograms of the indirect calculation of the 1RM with weight plates for each intensity. The adjustable strut supported the barbell aligned with the upright position of the participant and was placed on top of the digital scale. Depending on the kilograms that the digital scale indicated, the color (i.e., tension) of the elastic bands was modified. A horizontal band indicated the deepest position (i.e., 90° of knee flexion measured with a goniometer) for each participant (Fig. 1). This procedure was adopted to prevent the participants from going deeper than 90° of knee flexion. The execution tempo consisted of a 2-s eccentric phase until contacting (midthigh) the horizontal band and then a maximum-speed concentric phase.

#### 2.4.2 1RM calculation

The 1RM was indirectly calculated through the repetitions to the (technical) failure method with submaximal loads with weight plates. The participants started performing 20 repetitions using the bar (20 kg) with no extra weight. This was followed by a set performing 15 out of 20 possible repetitions, and a set performing 10 out of 13 possible repetitions. The fourth set consisted of a load that allowed performing from 8 to 12 repetitions. With the repetitions performed in the fourth set, Epley's formula (1RM = [1 + 0.033 × reps.] × kg) was used when ≥10 repetitions were performed [34] or O'Connor's formula (1RM = [1 + 0.025 × reps.] × kg) when <10 repetitions were performed [35]. A 5-min rest was permitted.

#### 2.4.3 Heart rate

Chest strap Heart rate monitors (Polar FT1, Polar Electro, Tampere, Finland) were used. The heart rate was registered at the end of the last repetition (i.e., 10% velocity loss) of each set.

#### 2.4.4 Movement velocity and maximum power

A linear position transducer (Speed4Lift, Madrid, Spain) with a coefficient of variation [CV] = 2.61% and intraclass correlation coefficient [ICC] = 0.87 [33] was employed to measure the total number of repetitions, the MPV and, P_MAX_ in each set. The P_MAX_ values were calculated using the weight provided by the elastic bands at the standing position.

#### 2.4.5 Perceived exertion values measurement

The RPE of the active muscles was obtained with the Resistance Intensity Scale for Exercise [12,36]. Participants were briefed on how to use the scale. Participants had to answer, on a scale of five points (easy, low, moderate, hard, maximal), “How hard do you feel your muscles working?” Participants were asked to exert the maximum intentional velocity in each repetition and were asked to report their RISE word descriptor immediately after the first and last repetition [37].

#### 2.4.6 Relative strength

The relative strength ratio was calculated as 1RM load (in kilograms)/body mass (in kilograms) [38]. In our study, all the volunteers showed normal to high relative strength levels (>1.0).

### 2.5 Statistical analyses

We used commercial software (SPSS, version 26.0, SPSS Inc., Armonk, NY, USA) to perform statistical analyses. The Shapiro–Wilk test was used to check the normality of the variables. Except for the number of repetitions performed at 55% 1RM and the RISE, all the variables presented a normal Gaussian distribution. A parametric analysis of variance (ANOVA) was used as it is robust [39]. Results are reported as mean and standard deviation (SD). A *p-*value <0.05 was uniformly established as a significance criterion.

A two-way mixed analysis of variance (ANOVA) assessed the effect of each %1RM (40%, 55%, 70%, and 85% 1RM), and relative strength level (medium [1.0–1.5] and high [>1.5]) on the number of repetitions performed, load, and heart rate. Besides that, a three-way mixed ANOVA was performed to evaluate the influence of the %1RM (40–85% 1RM), repetition (first repetition and last repetition), and relative strength level (medium [1.0–1.5] and high [>1.5]) on the MPV, P_MAX_, and RISE. Partial eta squared (η_p_^2^) was calculated as a measure of the effect size, and interpreted as low (<0.04), moderate (0.04–0.13), and large (>0.13). Planned pairwise comparisons were performed with the Bonferroni correction.

Furthermore, a categorical linear regression analysis was run to evaluate the potential of the RISE to potentially predict the external load parameters included in the study.

Finally, the between-session relative (ICC, model 3.1) and absolute (CV) reliability of each variable were assessed. ICC values were interpreted as poor (<0.50), moderate (0.50–0.75), good (0.75–0.90), and excellent (>0.90) reliability, based on the lower bound 95% confidence interval [40]. The CV was calculated by dividing the standard error of measurement by the participants’ mean score × 100. The standard error of measurement is the standard deviation of the difference between the two measurements divided by the square root of the number of measurements per subject [41]. It was defined as excellent reproducibility with a CV ≤ 10%, good reproducibility with a CV between 10 and 20%, acceptable with a CV between 20 and 30%, and poor reproducibility with a CV> 30% [42].

## 3 Results

### 3.1 Criterion variables for concurrent validation

In this subsection, the RISE is analyzed altogether with different variables that indicate the training load from a mechanical (MPV, P_MAX_, weight used, number of repetitions) and physiological (heart rate) perspective. Descriptive results of the RISE and the rest of the criterion variables at 40%–85% 1RM are shown in Table 1. Table 2 presents the same variables differentiating by the relative strength of the subjects. There were seven participants with normal relative strength (1.0–1.5), and eleven with relative strength levels considered as high (>1.5) [38].

**Table 1:** Concurrent variables to validate the perceived exertion (RISE) for velocity-based training.

Variables		40% 1RM	55% 1RM	70% 1RM	85% 1RM
RISE [[1], [2], [3], [4], [5]]	First repetition	1.17 (0.38)	1.78 (0.73)^1^	2.56 (0.70)^1,2^	3.11 (0.90)^1,2,3^
	Last repetition	2.28 (0.96)*	2.67 (0.69)*	3.17 (0.51)*^1,2^	3.67 (0.59)*^1,2,3^
MPV (m/s)	First repetition	0.94 (0.13)	0.90 (0.09)^1^	0.80 (0.10)^1,2^	0.73 (0.09)^1,2,3^
	Last repetition	0.82 (0.12)*	0.78 (0.09)*	0.69 (0.10)*^1,2^	0.63 (0.08)*^1,2,3^
P_MAX_ (W)	First repetition	408.83 (150.95)	530.22 (184.01)^1^	591.49 (187.40)^1,2^	649.60 (219.79)^1,2,3^
	Last repetition	355.34 (132.63)*	461.06 (161.20)*^1^	514.73 (168.61)*^1,2^	568.31 (189.90)*^1,2,3^
Weight (kg)		42.72 (12.15)	58.47 (16.25)^1^	74.39 (20.87)^1,2^	89.15 (25.62)^1,2,3^
Repetitions (number)		16.22 (3.57)	12.33 (2.03)^1^	8.39 (1.88)^1,2^	6.00 (1.91)^1,2,3^
Heart Rate (bpm)		155.18 (14.64)	154.76 (13.34)	153.76 (13.46)	150.12 (11.94)^1,2^
Results are presented as mean and standard deviation in parenthesis. ^1-4^: Statistically significant difference compared to those performed with the perceptual value indicated by that number (i.e., 1: 40%1RM; 2: 55%1RM; 3: 70%1RM; 4: 85% 1RM). * Very significant differences (p < 0.01) with the first repetition in each intensity. MPV: mean propulsive velocity. 1RM: one-repetition maximum. P_MAX_: maximum power. W: Watt. M/s: meters per second. Bpm: beats per minute. The perceived exertion is measured through the Resistance Intensity Scale for Exercise, with numerical values [[1], [2], [3], [4], [5]]: 1 = easy; 2 = low; 3 = moderate; 4 = hard; 5 = maximal).

**Table 2:** Concurrent variables to validate the perceived exertion (RISE) for velocity-based training when relative strength was considered.

Variable		40% 1RM		55% 1RM		70% 1RM		85% 1RM	
		High	Normal	High	Normal	High	Normal	High	Normal
RISE [[1], [2], [3], [4], [5]]	First rep	1.09 (0.30)	1.29 (0.49)	1.64 (0.81)	2.00 (0.58)^1^	2.45 (0.82)^1,2^	2.71 (0.49)^1^	2.91 (1.04)^1,2^	3.43 (0.53)^1,2^
	Last rep	2.09 (0.83)*	2.57 (1.13)*	2.64 (0.81)*	2.71 (0.49)*	3.18 (0.60)*	3.14 (0.38)^1^	3.64 (0.67)*^1,2^	3.71 (0.49)^2^
MPV (m/s)	First rep	1.02 (0.55)	0.83 (0.14)^†^	0.95 (0.52)^1^	0.82 (0.10)^†^	0.83 (0.09)^1,2^	0.75 (0.10)^1^	0.75 (0.09)^1,2,3^	0.69 (0.10)^1,2,3^
	Last rep	0.88 (0.56)*	0.72 (0.12)*^†^	0.82 (0.54)*^1^	0.72 (0.10)*^†^	0.72 (0.09)*^1,2^	0.65 (0.11)*^1,2^	0.65 (0.08)*^1,2,3^	0.60 (0.09)*^1,2,3^
P_MAX_ (W)	First rep	498.02 (92.51)	268.68 (113.15)^†^	635.55 (124.70)^1^	364.70 (133.10)^†,1^	695.75 (110.77)^1^	427.66 (166.76)^†1^	762.72 (154.21)^1,2,3^	471.84 (192.13)^†,1,2^
	Last rep	428.80 (93.38)*	239.91 (99.34)*^†^	550.18 (113.58)*^1^	321.01 (121.19)*^†,1^	607.93 (98.32)*,^1^	368.28 (153.29)*^†1^	666.63 (131.54)*^1,2,3^	413.81 (166.67)*^†,1,2,3^
Weight (kg)		49.20 (10.00)	32.43 (8.10)^†^	67.60 (12.59)^1^	44.14 (10.86)^†1^	85.75 (16.42)^1,2^	56.29 (14.28)^†,1,2^	102.25 (21.71)^1,2,3^	68.00 (17.20)^1,2,3^
Reps		14.90 (2.64)	17.71 (4.35)^†^	11.40 (0.97)1	13.14 (2.41)^†^	7.40 (1.65)^1,2^	9.43 (1.40)^†,1,2^	5.70 (1.83)^1,2^	6.14 (2.12)^1,2,3^
Heart Rate (bpm)		155.60 (17.88)	154.57 (9.55)	156.10 (16.58)	152.86 (7.45)	153.20 (16.43)	154.57 (8.79)	152.10 (14.56)	147.29 (6.80)^†2^
Results are presented as mean and standard deviation in parenthesis. High: subjects with high relative strength. Normal: subjects with normal relative strength. ^1-4^: Statistically significant difference compared to those performed with the perceptual value indicated by that number (i.e., 1: 40%1RM; 2: 55%1RM; 3: 70%1RM; 4: 85% 1RM). * Very significant differences (p < 0.01) with the first repetition in each intensity. † Significant differences (p < 0.05) in the same intensity when normal relative strength subjects were compared to their higher counterparts in each variable. MPV: mean propulsive velocity; P_MAX_: maximum power. The perceived exertion is measured through the Resistance Intensity Scale for Exercise, with numerical values [[1], [2], [3], [4], [5]]: 1 = easy; 2 = low; 3 = moderate; 4 = hard; 5 = maximal).									

All the concurrent variables, including the RISE, significantly varied as the %RM was modified (RISE: F_(3,51)_ = 52.64, *p* < 0.001, η_p_^2^ = 0.76; number of repetitions: F [3,45] = 78.26, *p* < 0.001, η_p_^2^ = 0.84; weight used: F_(1.16,17.45)_ = 233.16, *p* < 0.001, η_p_^2^ = 0.94, MPV: F_(2.015,34.248)_ = 67.50, *p* < 0.001, η_p_^2^ = 0.80; P_MAX_: F_(1.745, 29.661)_ = 95.22, *p* < 0.001, η_p_^2^ = 0.85; heart rate: F [3,45] = 5.63, *p* = 0.002, η_p_^2^ = 0.27).

When the relative strength was considered (%1RM*relative strength), the parameters related to the intensity were significantly modified according to the relative strength of the participants (weight used: F_(1.16,17,45)_ = 9.13, *p* = 0.006, η_p_^2^ = 0.38; MPV: F [3,48] = 7.89, *p* < 0.001, η_p_^2^ = 0.33). Considering that there was a preestablished homogeneous volume of work (i.e., 10% velocity loss), repetitions (*p* = 0.44), heart rate (*p* = 0.18), P_MAX_ (*p* = 0.19), and RISE (*p* = 0.88) were not modified.

Finally, the relative strength of the participants influenced the reduction of the MPV (F [1,16] = 8.85, *p* = 0.009, η_p_^2^ = 0.36) and P_MAX_ (F [1,16] = 20.96, *p* < 0.001, η_p_^2^ = 0.57) from the first to the last repetition. However, this did not happen with the RISE (*p* = 0.38).

### 3.2 Intersession reliability of the resistance intensity scale for exercise

Good to excellent (ICC = 0.77–0.99) relative reliability and acceptable to excellent absolute reliability (CV = 4.09%–20.23%) were observed. More specifically, all the parameters presented significant reliability results (*p* < 0.001) (weight used: ICC = 0.99, CV = 4.09%; number of repetitions: ICC = 0.79, CV = 18.16%; heart rate: ICC = 0.97, CV = 4.12%; MPV of the first repetition: ICC = 0.93, CV = 6.57%; MPV of the last repetition: ICC = 0.96, CV = 6.81%; P_MAX_ of the first repetition: ICC = 0.99, CV = 8.46%; P_MAX_ of the last repetition: ICC = 0.99, CV = 8.00%; RISE score of the first repetition: ICC = 0.81, CV = 20.23%; and RISE score of the last repetition: ICC = 0.77, CV = 17.45%.

### 3.3 Prediction of the intensity through resistance intensity scale for exercise

Table 3 presents the results of the categorical linear regression. In this regard, a significant (*p* < 0.001) linear association between RISE (independent variable), and the dependent variables (weight used, MPV, and number of repetitions at the first and last repetition) was found. Table 4 shows the equivalences of the dependent variables with each RISE score.

**Table 3:** Results of the linear regression. Predicting the external load through the Resistance Intensity Scale for Exercise.

		r	R^2^ (SEE)	p	Regression equation
Weight (kg)	First repetition	0.54	0.29(21.92)	<0.001	36.87+(13.62 × RISEscore)
Repetitions (number)	First repetition	0.61	0.37(3.66)	<0.001	16.70+(-2.77 × RISEscore)
MPV (m/s)	First repetition	0.63	0.40(0.10)	<0.001	1.02+(-0.08 × RISEscore)
	Last repetition	0.43	0.18(0.11)	<0.001	0.91+(-0.06 × RISEscore)

SEE: Standard error of estimation. RISEscore: Resistance Intensity Scale for Exercise's scores: 1 = easy, 2 = low, 3 = moderate, 4 = hard, 5 = maximal.

**Table 4:** Equivalences of the Resistance Intensity Scale for Exercise with the weight, repetitions, and mean propulsive velocity (MPV) derived from the regression analysis.

Resistance Intensity Scale for Exercise	*Easy*	*Low*	*Moderate*	*Hard*	*Maximal*
Weight (kg)	50.49	64.11	77.73	91.35	104.97
Repetitions (number)	13.93	11.16	8.39	5.62	2.85
MPV (first repetition)	0.94	0.86	0.78	0.70	0.62
MPV (last repetition)	0.85	0.79	0.73	0.67	0.61

## 4 Discussion

The aim was (i) to examine the concurrent validity, (ii) to analyze the between-session reliability of the RISE to quantify the intensity in squats performed at maximum velocity with elastic bands, and (iii) to test the RISE as a predictor of the weight, number of repetitions, and MPV (external load parameters). The novel findings highlight the validity and reliability of the RISE to quantify the load during VBT with elastic bands along the 1RM continuum. Additionally, the RISE allowed the prediction of the external load parameters analyzed.

The RISE has been previously validated for healthy middle [36] and older adults [12] through changes in perceived exertion between different resistance intensity levels (%RM) [43]. However, the validation was set at a moderate pace and the RISE score was given after the set. Interestingly, the novel study found differences in the study variables (RISE and concurrent variables) at different loads with a between-load difference as small as 15%1RM. Moreover, only one repetition was needed to find these differences. This method of obtaining the perceived effort only in the first repetition of the set had already been demonstrated using numerical descriptors (i.e., OMNI-Resistance Exercise Scale for elastic bands) in healthy young adults [23,27].

Regarding the heart rate, it did not show significant changes. This could be probably due to the short duration of the high-intensity muscle contractions (the set finished at 10% velocity loss), which do not require sustained elevated cardiac output [44]. In contrast, previous validation studies did report heart rate significant changes [12,36,43] due to the greater number of repetitions. These findings confirm that a perceived exertion scale with verbal descriptors, such as the RISE, is a sensitive tool to evaluate differences in the load across the RM continuum. This is in line with the first hypothesis of the study.

Additionally, the RISE and the rest of the concurrent variables were analyzed considering the relative strength of the participants. In this regard, the RPE measured through the RISE was not affected by the participants’ relative strength, while some of the criterion variables (e.g., MPV, weight used, P_MAX_) were. This finding means that RISE scores are not different between participants with high relative strength and normal relative strength. Therefore, the RISE can be used independently from the participants' relative strength. In agreement, previous studies have shown that perceived exertion is a valid, reliable, and easy-to-use method for different population groups to quantify intensity during variable resistance training at constant velocity [12,36]. Added to our results, the RISE appears as a good indicator of the intensity at maximum speeds, independently of the physical condition level (relative strength) of the participants.

As for the second hypothesis, we obtained good to excellent ICC and acceptable to excellent CV on the mechanical (MPV, P_MAX_, weight used, and repetitions) and physiological (heart rate) criterion variables along with the validated variable (RISE). Therefore, the RISE is a reliable tool to quantify the training intensity for VBT with elastic bands at different loads. Although some studies suggest that programming based on objective methods (e.g., MPV) generates greater gains in the 1RM compared to subjective methods (e.g., perceived exertion) [45], the combination of both subjective (i.e., perceived exertion) and objective (e.g., velocity) methods to quantify the intensity is reliable, providing superior strength results compared to training exclusively based on %1RM [46]. In this regard, our results showed that perceived exertion (i.e., RISE) is a useful tool to quantify training intensity, which is in line with Helms et al. [9].

A previous study [47] reported a high standard error estimation between the Borg scale (recorded at the second repetition) and the maximum repetitions (more than 45%) at different intensities (i.e., 50–90% 1RM), which prevented them to perform estimation equations. However, they did not report the speed of execution. The values obtained in the present study with the categorical linear regression endorse the use of the estimation equations employed. This supports the use of the first- and last-repetition RPE as a satisfactory strategy for monitoring the number of repetitions, weight used, and MPV during VBT with elastic bands at different loads. Similarly, previous models to predict the relative load from the MPV and RPE demonstrated high accuracy (i.e., 79% and 86%, respectively) in the parallel squat with constant load [30]. Similar to our results, Lagally et al. [48] asked the participants to lift a load that elicited 3, 6, and 9 on an RPE scale (OMNI-RES) in two exercises. The average %1RM lifted demonstrated that the RPE score was an accurate and reliable tool for selecting intensities. However, they did not evaluate the RISE. Therefore, to the best of our knowledge, this is the first study that analyses the potential of the RISE to predict the movement speed in the parallel squat with elastic bands in resistance-trained subjects.

### 4.1 Limitations

The study is not free of limitations. Firstly, it is relevant to validate the association between the RISE and MPV in different population profiles (e.g., older adults, adolescents, worse physical condition), exercises (i.e., upper limbs), and elastic bands models. Second, despite the physical activity levels being moderate to high in all participants, the resistance training experience and the modality of sport practiced were not reported, which could influence the results [49]. Therefore, the findings should be taken with caution regarding individuals highly trained in resistance training. Third, there are differences between variable resistances (e.g., elastic bands) and constant resistances [49]. Considering that the load for each participant was programmed according to their %RM with weight plates, the weight with elastic bands was equal only at the upright position of the squat. Therefore, future studies may evaluate a method to obtain the pertinent load to use with an RM test with elastic bands. Finally, we did not study possible perceptual differences between sexes. However, this kind of scale is valid for both sexes when isotonic muscle actions are performed at identical relative intensities [11].

### 4.2 Practical applications

The RISE is a valid, reliable, cost-effective, time-efficient, and easy-to-use tool for monitoring VBT with elastic bands. It has been shown that the RISE allows quantifying the intensity both in the first and last repetition of a given resistance training set. This can be extrapolated as two key points.

First, the score of the first repetition on the RISE may serve to adjust and modify the resistance early in each set. In this regard, if the first-repetition RISE score is not above or below the desired exercise stimuli the set can be stopped in the first repetition, avoiding excessive fatigue.

Second, considering that the MPV, number of repetitions, and weight used decrease when the RPE scores increase through the 1RM continuum, the RISE scores perceived by the participant can help to stop the set at the appropriate moment (e.g., to end the set at 10% velocity loss), optimizing the adaptations and avoiding undue stress.

## 5 Conclusion

The study aimed to examine the concurrent validity and reliability of the RISE to quantify the load during squats with elastic bands. Additionally, we evaluated the potential of the RISE to predict external load parameters at different loads (i.e., 40–85%1RM). The novel findings highlight the appropriateness and reliability of the RISE for monitoring VBT with elastic bands along the 1RM continuum. Additionally, the RISE allowed the prediction of the number of repetitions, weight, and the MPV. Coaches and practitioners could use the score of the first and last repetition on the RISE to adapt the load and stop the set earlier, therefore, optimizing the adaptations and avoiding undue stress.

## Funding

This research did not receive any specific grant from funding agencies in the public, commercial, or not-for-profit sectors.

## Data availability statement

All data generated or analyzed during this study are included in this published article. The databases are available upon reasonable request to the corresponding author.

## CRediT authorship contribution statement

**Juan C. Colado:** Writing – review & editing, Visualization, Supervision, Resources, Project administration, Methodology, Conceptualization. **Javier Gene-Morales:** Writing – review & editing, Writing – original draft, Methodology, Investigation, Data curation, Conceptualization. **Pablo Jiménez-Martínez:** Writing – review & editing. **Angel Saez-Berlanga:** Writing – review & editing, Methodology, Investigation. **Ana María Ferri-Caruana:** Software, Resources, Methodology, Investigation. **Amador Garcia-Ramos:** Writing – review & editing, Resources. **Jorge Flandez:** Writing – review & editing. **Carlos Babiloni-Lopez:** Writing – review & editing, Writing – original draft, Validation, Formal analysis.

## Declaration of competing interest

This research is part of a larger project (approved by the Ethics Committee of the University of Valencia; code: H20190325095509; previously published studies [23,24]) that aims to analyze the outcomes of using elastic bands, instead of weight plates, to load the bar in the parallel squat at maximum velocity of execution and validate the RISE of the first and last repetition. The authors declare that there is no conflict of interest. Amador Garcia-Ramos is an Academic Editor for Heliyon.
